# Comparison of the Content of Several Elements in Seawater, Sea Cucumber *Eupentacta fraudatrix* and Its High-Molecular-Mass Multiprotein Complex

**DOI:** 10.3390/molecules27061958

**Published:** 2022-03-17

**Authors:** Natalia P. Zaksas, Anna M. Timofeeva, Pavel S. Dmitrenok, Svetlana E. Soboleva, Georgy Nevinsky

**Affiliations:** 1 Institute of Automation and Electrometry, Siberian Division of Russian Academy of Sciences, Pr. Academician Koptyug 1, Novosibirsk 630090, Russia; natzaksas@gmail.com; 2 Institute of Chemical Biology and Fundamental Medicine, Siberian Division of Russian Academy of Sciences, Pr. Akademika Lavrentieva 8, Novosibirsk 630090, Russia; bezukaf@mail.ru (A.M.T.); sb543@ngs.ru (S.E.S.); 3 G. B. Elyakov Pacific Institute of Bioorganic Chemistry, Far Eastern Brunch of the Russian Academy of Sciences, 159 Pr. 100 let Vladivostoku, Vladivostok 690022, Russia; paveldmt@piboc.dvo.ru

**Keywords:** seawater, sea cucumbers *Eupentacta fraudatrix*, high-molecular-mass multiprotein complex, major and trace elements

## Abstract

Metal ions and other elements play many different critical roles in all biological processes. They can be especially important in high concentrations for the functioning of organisms living in seawater. It is important to understand how much the concentrations of different trace elements in such organisms can be higher than in seawater. Some marine organisms capable of rapid recovery after different injuries are fascinating in this regard. Sea cucumbers *Eupentacta fraudatrix* can completely restore all organs and the whole body within several weeks after their division into two parts. Here, for the first time, a comparison of the content of different elements in seawater, sea cucumber, and its very stable multiprotein complex (2000 kDa) was performed using two-jet plasma atomic emission spectrometry. Among the 18 elements we found in sea cucumbers, seawater contained only six elements in detectable amounts, and their content decreased in the following order: Mg > Ca > B > Sr ≈ Si > Cr (0.13–930 µg/g of seawater). The content of these elements in sea cucumbers was higher compared with seawater (-fold): Ca (714) > Sr (459) > Cr (75) > Si (42)> B (12) > Mg (6.9). Only four of them had a higher concentration in the protein complex than in seawater (-fold): Si (120.0) > Cr (31.5) > Ca (9.1) > Sr (8.8). The contents of Mg and B were lower in the protein complex than in seawater. The content of elements additionally found in sea cucumbers decreased in the order (µg/g of powder) of P (1100) > Fe (47) > Mn (26) > Ba (15) > Zn (13) > Al (9.3) > Mo (2.8) > Cu (1.4) > Cd (0.3), and in the protein complex, in the order of P (290) > Zn (51) > Fe (23) > Al (14) ≈ Ni (13) > Cu (7.5) > Ba (2.5) ≈ Co (2.0) ≈ Mn (1.6) > Cd (0.7) >Ag (0.2). Thus, sea cucumbers accumulate various elements, including those contained in very low concentrations in seawater. The possible biological roles of these elements are discussed here.

## 1. Introduction

Major and trace elements, including metal ions, have influential roles in many processes in all living organisms, including regular and pathological conditions [1,2,3,4,5,6]. They take part in the transport of gases and nutrients, maintaining temperature, acid–base balance, homeostasis of the living organisms, functioning of various enzymes, proteins, DNA cytoskeleton activation, etc. Living organisms cannot exist without some elements, including metal ions, participating in a variety of biological processes.

Today, there are many methods for analyzing different elements in various biological samples. Inductively coupled plasma atomic emission and atomic absorption spectrometry usually require matrix destruction with concentrated acids to study animal tissues [7,8]. Two-jet plasma atomic emission spectrometry (TJP-AES) allows direct analysis of powdered samples due to a high-power excitation source. Currently, TJP-AES, developed in the mid-1970s [9], has been significantly improved due to the use of a new modern plasmatron designed at VMK-Optoelektronika (Russia).

A photograph of the plasma torch and a scheme of the electrode unit are shown in Supplementary Figure S1 (in Supplementary Materials). An automatic sample-introduction system allows acquiring reproducible results at powder sample introduction [10,11]. First, TJP-AES was used to directly analyze geological samples after little or no chemical pretreatment [10]. All the features of this method and ways to use were previously described in more detail [11]. Later, this method was applied for multi-elemental analysis of many elements in dried and carefully powdered different animal organs [12,13,14,15]. Due to the weak matrix effects in the plasma, it turned out to be possible to use unified calibration samples based on graphite powder to analyze completely different samples.

The possibility of analysis of small amounts of samples (several mg) allows the use of TJP-AES in the case of biomedical experiments with small animals. It was shown that the content of different elements in mice plasma of the control group of 2-month-old *balb/c* mice decreased in the following order: Ca > Mg > Si > Fe > Zn > Cu ≥ Al ≥ B [16]. Using TJP-AES, the mass percent of metal ions in the lyophilized samples of plasma of healthy Wistar rats was estimated: Ca > Mg > Fe > Cu ≥ Zn > Al ≥ Sr > Ti ≥ Mo ≥ Mn ≥ Pb ≥ Co ≥ Ni > Ag [17]. Blood preparations of Wistar rats were used to purify homogeneous IgGs [18]. In contrast to plasma preparations, IgGs from Wistar rat plasmas did not contain detectable amounts of Ti, Mo, Si, Cr, or Ag: Fe ≥ Pb ≥ Zn ≥ Cu ≥ Al ≥ Ca ≥ Ni ≥ Mn > Co ≥ Mg [17]. We obtained the milk powders by using fresh mother’s milk and analyzed them using TJP-AES, and their concentrations increased in the following order: Ca > P > Mg > Al > Zn > Fe > Cu > B > Ni ≈ Cr ≈ Ba ≈ Pb > Sr ≈ Cd > Mn > Ag [18]. Many various biological processes are carried out by protein complexes [19,20]. This was recently shown the existence of very stable multi-protein complexes (SPCs; ~1000 ± 100 kDa) in breast milk [21], human placentas [22,23], and sea urchin eggs [24]. These very stable multi-protein complexes contain various major and minor proteins with high, moderate, and low molecular masses [23,24]. These complexes dissociate only in the presence of 8.0 M urea containing 1.0 M NaCl and EDTA.

Very stable protein complexes are likely to exist in different biological liquids of various organisms. At the same time, in the literature, there are no data concerning the possible type of elements and their role in the formation and stabilization of the protein complexes, as well as their role in catalytic activities. TJP-AES was used first to compare the content of different metals in human milk and stable protein complexes from the same milk [18]. It has been shown that the complex accumulates different metals from milk to varying degrees. The content of various elements in the protein complex was higher than in milk (-fold): Sr (110.0) > Mn (82.0) > Ni (38.0) > Ag (28.7) > B (19.7) > Cu (12.7) > Zn (9.6) > Cd (9.0) > Cr (6.2) > Ba (5.8) > Pb (4.0) > Fe (3.8) > Al (3.0) > P (2.6) > Ca (1.2) ≈ Mg (1.2) [18]. It was shown that, in the presence of 8 M urea, the efficiency of the complex dissociation strongly increases after the addition of EDTA. This indicates that metal-dependent interactions are formed in addition to hydrogen bonds between the complex components. With this in mind, the analysis of stable complexes with extended biological functions is of particular interest. In marine organisms capable of rapid regeneration, unique, highly stable complexes can exist.

We recently isolated a highly stable complex from sea cucumber *E. fraudatrix* [25]. It was shown that in contrast to human milk, placenta, and sea urchin, which have complexes of ~1000 ± 100 kDa, the sea cucumber complex is about 2000 kDa. It contains ~15 major and many minor proteins and peptides with molecular masses (MMs) of 2.0–9.0 kDa [25]. The complex dissociates effectively only in the presence of 3.0 M MgCl_2_, destroying immunocomplexes, while its best breakdown occurs in the presence of 8.0 M urea supplemented with 0.1 M EDTA. Our data show that a very stable protein complex formation occurs mainly due to the combination of molecular interactions formed by metal ions and hydrogen bonds [25]. Taking this into account, it was interesting to analyze which of the metal ions of the seawater are stockpiled by sea cucumbers and which are accumulated by the stable complex.

In this work, we carried out for the first time the determination of the content of 18 elements in seawater, sea cucumber organisms, and the protein complex using the TJP-AES method. Such analysis is important for understanding the possible level of accumulation of various metal ions by sea cucumbers, which are important for the functioning of their internal components.

## 2. Results

### 2.1. Seawater, Cucumbers, and Multi-Protein Complex

Seawater and cucumbers *Eupentacta fraudatrix* were collected from the same place in the Japanese Sea (Peter the Great Bay) using special plastic Corning 50 mL polypropylene vessels. All analyses were carried out under special conditions free of any bacterial and viral contaminants, pathogens, and other possible contaminants. The preparation procedure of homogenates of sea cucumbers was described earlier [25]. After removing insoluble components by centrifugation and dialysis, the supernatant was concentrated and subjected to fast protein liquid chromatography (FPLC) gel filtration on Sepharose 4B, as in a previous paper [25]. The isolation of a very stable complex was previously described in detail [25]. A typical gel filtration profile is shown in Supplementary Figure S2A (in Supplementary Materials). One protein peak (~2000 kDa) separates well from other different proteins. Repeated gel filtration of the complex on the Sepharose 4B demonstrated only one peak with the same MM, ~2000 kDa (Supplementary Figure S2B). Using the light scattering method, it was shown that the complex dissociates only under conditions of destruction of strong complexes at a very high salt concentration (3.0 M MgCl_2_) or 8.0 M urea in the presence of 0.1 M EDTA (Supplementary Figure S3) [25]. The data obtained indicate that a strong complex is formed between its components mainly due to the formation of hydrogen bonds (destroyed by 8.0 M urea), electrostatic contacts (destroyed by 3.0 M MgCl_2_), and contacts with metal ions (destroyed by EDTA). The obtained preparations of sea cucumber organisms and the high molecular weight complex were thoroughly lyophilized, ground to a powder, again dried by lyophilization, and used for analysis.

### 2.2. Determination of Elements by TJP-AES

TJP-AES allows using a small sample to simultaneously estimate the content of about 20 different elements [11,12,13,14,15,16,18]. Analysis of seawater was carried out after evaporation of water aliquot on graphite powder. Sea cucumbers and the protein complex were previously carbonized, ground in a Plexiglas mortar, and then diluted with a spectroscopic buffer (graphite powder, containing 15 wt % NaCl). Final powder samples of sea cucumbers and the protein complex were used to analyze different elements by TJP-AES, as in previous papers [11,12,13,14,15,16,18]. The data are presented as µg of found elements per gram of the samples (Table 1). It should be mentioned that the contents of some trace elements in seawater are below the detection limits of the technique, which is not sensitive enough for this matrix.

Seawater contained only six reliably tested elements in comparatively high concentrations, which decreased in the following order: Mg > Ca > B > Sr ≈ Si > Cr (Table 1). Some elements, including metals, the content of which in the seawater was low, efficiently accumulated in the organisms of the sea cucumbers. The order of increasing concentrations of six elements in sea cucumbers, found in seawater in reliably identifiable concentrations, was different compared to seawater: Ca > Mg > Sr > Si ≈ B > Cr (Table 1). At the same time, the level of the increase in the content of these elements in sea cucumber organisms compared to that for seawater was very different (-fold): Ca (714.3) > Sr (458.8) > Cr (74.6) > Si (42.0) > B (12.2) > Mg (6.9) (Table 1). Interestingly, the increase in magnesium content, which was contained in the seawater in the highest concentration (930.0 μg/g), was minimal in the sea cucumbers, 6.9-fold. However, the calcium content, the concentration of which in seawater was also high (350.0 μg/g), strongly increased in sea cucumbers, by 714.3-fold.

Several elements were not determined in seawater since their concentrations were lower than the detection limits of the method. The detection limits of elements in seawater vary in the range 0.003–0.5 μg/g (Table 1). Taking this into account, the possible level of increase in the content of some elements was assessed in accordance with the corresponding limits of their detection. Finally, this approach made it possible to estimate the approximate lower limit of the increase in the content of some elements in sea cucumber compared with seawater.

Sea cucumber and its stable protein complex contained 18 reliably detectable elements (Table 1). The content of three elements (Ni, Co, and Ag) in sea cucumber was lower than detection limits. These elements (Ni > Co >Ag) were found only in the stable protein complex, but the complex contained Mo on a level lower than the detection limit (<2 μg/g) (Table 1).

As expected, the order of increase in the content of some elements in the complex compared with seawater differs from that for the whole organism of sea cucumber and seawater. In the case of elements quantified in seawater, four elements demonstrate an increase in the content in both the complex and the organisms of sea cucumbers compared with seawater (-fold for the complex and whole organism): Si (120.0 and 42.0), Sr (8.8 and 458.8), Cr (31.5 and 74.6), and Ca (9.1 and 714.3) (Table 1). Interestingly, in the case of two other elements, the content in the whole organism of sea cucumbers is higher than in seawater (B, 12.2-fold and Mg, 6.9-fold). In contrast, the amount of B in the protein complex is nearly the same as in seawater, but the content of Mg is 21.1-fold lower (Table 1).

As noted above, Mo was detected only in the whole organism of sea cucumbers, while Ag, Co, and Ni were above the detection limits only in the protein complex.

## 3. Discussion

### 3.1. The Content of Metal Ions in Sea Cucumber

Metal ions and some other elements play a vital role in all living organisms. However, their content can be very different for creatures living on land, in rivers, and in seawater. In this paper, we first compared the content of various elements in seawater, sea cucumbers, and their very stable protein complexes.

The content of some macro- and microelements are already reported in humans and other mammals [26,27,28,29,30], some plants [31,32,33,34,35,36], and seagrass [37,38,39]. There is a lack of data in the literature on the content of trace elements in sea echinoderms, including sea cucumbers *E. fraudatrix.* The biological roles of various microelements have now been mainly described for humans, some animals, and plants. It should be assumed that, to one degree or another, the roles of various microelements in these organisms are close or similar to those in echinoderms, including sea cucumbers.

The content of some elements in seawater, which are found in sea cucumbers and their stable protein complexes, is so low that it was impossible to estimate the extent of changes in the level of their accumulation in the organisms of these echinoderms compared with seawater. However, all the elements determined in sea cucumbers presumably play an important biological role in the functioning of their organisms.

In this regard, it is important to estimate how much the contents of different elements in the organisms of sea cucumbers and their protein complexes increase in comparison with seawater. The concentration of elements found in seawater declines in the order Mg > Ca > B > Sr > Si > Cr (Table 1).

### 3.2. Biological Role of Different Metal Ions

Among these elements, the maximum increase (714.4-fold) in the content in organisms of sea cucumbers in comparison with seawater is observed for calcium. In the case of the protein complex, its content compared to seawater increases only 9.1 times (Table 1). Calcium is one of the most critical macroelements of all living organisms [26,27,28]. Calcium regulates the permeability of cell membranes, initiates cell responses to various external stimuli, and determines cell differentiation and muscle contraction, secretion, and peristalsis. It regulates the activity of many enzymes, controls the work of some endocrine glands, and has a desensitizing and anti-inflammatory effect. Calcium is one of the most essential macroelements of all living systems.

Strontium is in second place in terms of increasing concentration (458.8-fold) in organisms of sea cucumbers (Table 1). Strontium is mainly found in the bone tissue of 99% of analyzed mammalians [40]. Until now, the role of this element as a vital one has not been precisely established, but it has been proven that strontium plays an important role in the formation and strengthening of tooth enamel and in the processes of bone formation in animals. Strontium is an integral part of microorganisms and plants. Seaweed and terrestrial plants contain strontium. In small doses, Sr performs functions similar to those of calcium [40].

Chromium is also among the elements found in relatively high concentrations (0.13 μg/g) in seawater. A substantial increase in the content of chromium compared to seawater was found both in sea cucumbers (74.6-fold) and in the stable protein complex (31.5-fold) (Table 1). Chromium ions may be very important for forming stable complexes in various biological fluids of living organisms [30,41,42]. For example, the Cr content in the milk protein complex is 110 times higher than in milk [18]. Moreover, in the case of the milk complex, chromium takes first place in the range of the increase in its concentration compared to milk. Chromium ions are part of some enzymes that maintain normal glucose levels, regulate lipid metabolism, ensure the structural integrity of nucleic acids, regulate the thyroid gland, regulate the activity of the heart muscle and blood vessels, and also enhance regeneration processes, promoting the elimination of toxic elements from the body [30,41,42].

The silicon concentration in seawater is also relatively high (1.5 μg/g). The concentration of silicon in the protein complex was 120 times higher than in seawater, and in the whole organism, the concentration was 42 times higher than in seawater (Table 1). Silicon was shown to participate in the normal metabolism of higher animals [43]. It was found to play an important role in connective tissues, especially in cartilage and bone. Silicon was shown to be a major ion of osteogenic cells and is present in an increased amount in the metabolically active state of the cells; furthermore, it reaches a relatively high level in the cells’ mitochondria. In addition, silicon takes part in the biochemistry of the subcellular enzyme-containing structures and provides important interrelationships with other elements [43].

The boron content in sea cucumbers is 12.2 times higher than in seawater, but its concentration in the protein complex is comparable to seawater (Table 1). Interestingly, the average boron concentration in the complex preparations from milk is 19.7 times higher than in the milk itself [18]. Boron regulates the activity of parathyroid hormones, increases the absorption of calcium and magnesium and affects their metabolism, participates in the exchange of phosphorus, promotes the transition of vitamin D into an active form, reduces the risk of cancer, and increases the level of estrogen and testosterone in the blood [44,45].

The magnesium content in seawater is the highest. The magnesium content in sea cucumbers is only 6.9 times higher than in seawater (Table 1). At the same time, its concentration in the protein complex is 21 times lower than in seawater and 145.5 times lower than in the whole organism of sea cucumbers (Table 1). Magnesium ions are extremely important for all living organisms on Earth [26,27]. Magnesium is a cofactor of many enzymes (it takes part in the release of energy from food). This element plays a significant role in the transmission of nerve impulses and is necessary for the rhythmic work of the heart. Magnesium actively participates in the metabolism of protein and nucleic acids, regulates mitochondrial production and transfer of energy, regulates signal transmission in the nervous and muscle tissue, promotes relaxation of smooth muscle fibers, reduces platelet aggregation, and also accelerates the passage of intestinal contents [26,27].

It should be noted that the content of some elements in seawater is so low that it is not possible to accurately determine their concentration due to the detection limits of the method. Therefore, the increase in the content of these elements in the organisms of sea cucumbers can be estimated only approximately and somewhat conditionally, taking into account the detection limits for different elements. The content of some elements in seawater can be comparable to or lower than their detection limits. Nevertheless, it was interesting to estimate the lower limit of the increase in the content of such elements in sea cucumbers compared to seawater. Therefore, it should be taken into account that this parameter may be equal to or greater than (≥) the ratio found between content in the organism and the protein complex and detection limits for each element.

Considering this conditional rating evaluation, the increase Mn ions in sea cucumber compared to seawater may be ≥5200-fold (Table 1). The manganese content in the stable complex is also high, ≥320 times higher than in seawater. It should be noted that the stable complex from human milk contains 82-fold higher Mn concentration than milk itself [18], while the complex isolated from cucumbers has 16 times lower Mn concentration than an organism of cucumbers. The biological role of Mn ions is very multifaceted [46,47,48]. It is a part of many enzymes; it is a catalyst for some chemical reactions, including synthesis of nucleic acids, proteins, and neurotransmitters; participates in the exchange of hormones; prevents the oxidation of various compounds by free radicals; ensures the stability of cell membranes; and regulates the functioning of muscles and the development of connective tissue, cartilage, and skeleton [47,48].

In the same way (≥), the possible lower degree of other elements’ increases in sea cucumbers compared with seawater can be estimated. Of those elements, the content of phosphorus in seawater is very low. The organisms of sea cucumbers contain ≥2200-fold phosphorus, but in the stable protein complex, its concentration is ≥580 times higher than in seawater and 3.8 times lower than in sea cucumber organisms (Table 1). The increased content of phosphorus in whole sea cucumber organisms is evident. Phosphorus is a part of many body substances (phospholipids, phosphoproteins, nucleotides, coenzymes, enzymes, etc.). Phosphoric acid residues are part of nucleic acids and nucleotides and ATP and creatine phosphate, the most important accumulators and carriers of energy. Residues of phosphoric acid are also part of the buffer systems of living organisms, regulating their pH value [49].

Sea cucumbers contain an increased concentration of iron (≥940-fold higher than seawater), but its content in the stable complex is two times lower (Table 1). The iron content in the milk protein complex is about 3.8 times higher than in milk [18]. Iron is included in the group of essential (vital) microelements of living organisms. The biological role of iron in the body is significant—it participates in redox processes, growth and aging of tissues, mechanisms of immunity, hematopoiesis, supply of organs and tissues with oxygen, and the functioning of many enzymes [30,48,49,50].

It is somewhat unexpected that sea cucumbers contain an increased concentration of barium ions (15 µg/g), which may be ≥300-fold different compared with seawater (Table 1). However, the content of barium in the composition of the stable protein complex is about 6 times lower. Interestingly, barium ions are also included in the protein complex of human milk, in which their content is 5.8 times higher than in milk [18]. Barium is a toxic trace element. In living organisms, barium ions have a pronounced effect on smooth muscles. In trace amounts, it is found in all organs and tissues, but the highest concentration of this trace element is found in the brain, spleen, muscles, and also on the lens of the eye. About 90% of the entire trace element is concentrated in bones and teeth [51,52].

Increasing content (≥260-fold) in sea cucumber organisms compared to seawater was revealed for zinc (Table 1). The content of zinc ions in the protein complex is ≥1020 times higher than in seawater. The concentration of zinc ions in the milk protein complex is 9.6-fold higher than in milk [18]. Zinc takes part in the processes of cell division and differentiation, the formation of T-cell immunity, and the functioning of dozens of enzymes, including Zn-dependent metalloproteases, superoxide dismutases, and dihydrocorticosterone [30,53]. Zinc has an essential role in the processes of skin regeneration, hair and nail growth, and the secretion of the sebaceous glands. For sea cucumbers, it seems important that zinc supports reproductive function and participates in cell and tissue regeneration processes.

In terms of the increase in relative concentration compared to seawater, copper (Cu) ions in a complete organism are ≥200-fold, but it is significantly higher for protein complexes, ≥1071-fold (Table 1). The content of copper ions in a stable milk protein complex is 12.3 times higher than in milk [18]. Copper ions are components of many enzymes with redox activity; participate in iron metabolism; increase the absorption of proteins and carbohydrates; take part in providing tissues with oxygen; participate in the formation of connective tissue and bone growth; maintain the elasticity of the walls of blood vessels, alveoli, and skin; and have pronounced anti-inflammatory properties [37,54]. All these properties of copper ions are necessary for a vast number of living organisms.

It was unexpected that sea cucumbers (9.3 µg/g; ≥185 times in comparison with seawater) and their stable protein complexes (14.0 µg/g) contain the trace element aluminum in concentrations comparable to those of zinc and chromium, and greater than copper ions (1.4 and 7.5 µg/g) (Table 1). However, the same situation is observed for the stable protein complex from milk, in which the aluminum content is very high (124 µg/g), 3 times higher than in milk [18]. Aluminum participates in the formation of phosphate and protein complexes; the processes of the formation of the skeleton, cartilage, regeneration of bone, connective, and epithelial tissues; has inhibiting or activating effects on digestive enzymes, depending on the concentration; and is able to affect the function of the parathyroid glands [55,56].

The cadmium content in sea cucumbers differs ≥60-fold compared with seawater, and is about two times higher in the protein complex than in whole organism of sea cucumbers (Table 1). A similar situation is observed for milk; the protein complex containing this metal is 9.0 times more elevated than in milk [18]. Cadmium is toxic even at low concentrations and its effect on enzyme and carbohydrate metabolism is negative [57,58,59].

A unique trace element is molybdenum, which is found only in whole sea cucumbers in concentration ≥56 times in comparison with seawater but absent in their high-molecular-weight protein complex (Table 1). Molybdenum was also not found in the protein complex of milk [18]. This trace element is essential for living organisms. It participates in the metabolism of protein and purine bases, regulates the transformation of organic substances containing nitrogen, promotes the elimination of metabolic products from the body, neutralizes toxins, plays an important role in the formation of hemoglobin, improves the absorption and effects of vitamins, takes part in fat metabolism, helps to reduce glucose in the blood, facilitates the implementation of tissue respiration, and improves the function of the reproductive system [30,32].

Nickel (≥260-fold in comparison with seawater), silver (≥66.7-fold), and cobalt (≥66.7-fold) in reliably detectable amounts were found only in the protein complexes of sea cucumbers (Table 1). Interestingly, nickel and silver are found in the milk protein complex at 38.0- and 28.7-fold higher concentrations than in milk, while cobalt content was very low [30]. It seems somewhat odd that sea cucumber organisms contain nickel in low concentrations given the important biological role of nickel. Nickel is actively involved in the structural organization and functioning of the main cellular components—DNAs, RNAs, proteins, and enzymes. It carries out hormonal regulation of the body, is a potent stimulator of the formation of erythrocytes in the hematopoietic tissue of the bone marrow, facilitates hemoglobin synthesis, and increases the absorption of iron by the body [60,61]. The reduced nickel concentration in the organisms of sea cucumbers seems to be justified since it is toxic at high concentrations [60,61].

The content of silver in the protein complex may be ≥66.7 times higher than in seawater (Table 1). Silver in living organisms has pronounced bactericidal, antiseptic, anti-inflammatory, and astringent effects [62]. However, today, silver is not considered to be a vital ultra-microelement for the human body. Given this, it is not easy to imagine what role silver may play in the protein complexes of sea cucumbers and human milk. It is possible that the presence of silver in protein complexes may be due to the fact that this element can be present in the bodies of marine animals only because it was adsorbed or incorporated mechanically, without playing a biological role.

Cobalt was revealed only in the protein complex in concentrations approximately ≥66.7 greater than in seawater. Co plays an important role in the life of the body, as it stimulates the processes of hematopoiesis, participates in the synthesis of vitamin B12, regulates the functions of the nervous system, normalizes metabolism, stimulates the synthesis of proteins and the growth of bone tissue, and exhibits anti-sclerotic and immunostimulating effects [63].

## 4. Materials and Methods

### 4.1. Reagents

Extra pure NaCl (cat. S7653) and Tris (cat. T6791) were from Sigma-Aldrich (St. Louis, MO, USA). Sepharose 4B was from GE Healthcare Life Sciences (New York, NY, USA). Sea cucumber *E. fraudatrix* was collected from the Japan Sea (Peter the Great Bay). Samples of sea cucumbers were frozen to −70 °C and kept until the extracts’ preparation. Seawater samples were collected from the same place using sterile Corning CLS430829 50 mL CentrifugeTubes CentriStar Cap Polypropylene (Corning, Glendale, AZ, USA).

### 4.2. Purification of Stable Complexes by Gel Filtration

The preparation of homogenates of 10 sea cucumbers was carried out as described in a previous paper [25]. Homogenates were centrifuged at 17,000× *g* for 45 min two times and then filtered through a 0.1 nm filter, dialyzed four times against milli-Q water, and concentrated as in [25]. The supernatant was additionally centrifuged at 12,000× *g* 4 °C for 10 min and subjected to FPLC gel filtration on Sepharose 4B to obtain a very stable protein complex (2000 kDa), as in a previous paper [25]. Sepharose 4B was equilibrated in TBS (20 mM Tris HCl pH 7.5; 0.15 M NaCl) as in [25]. Before use, to remove possible metal ions, TBS buffer was passed through a Chelex-100 column. All eluted fractions (1 mL) were collected. The fraction corresponding to the complex was subjected to triple dialysis against milli-Q water.

### 4.3. Multi-Elemental Analysis of the Samples

Before removing possible bacterial contamination, seawater samples were filtered using Millex membranes (0.1 μm) For the analysis of seawater, 1 mL seawater was evaporated on 50 mg of high-purity graphite powder placed in a previously weighed quartz cup under an IR lamp. The dry remainder was weighed, ground in a Plexiglas mortar, and diluted with graphite powder 3 times. Then, a 20 mg sample (4 replicates) was introduced into the plasma.

Combined homogenates of the 10 sea cucumber preparations or stable protein complexes were thoroughly dried by lyophilization. Then, the dry samples were thoroughly rubbed, and the fine powders were additionally dried. Final powder samples were used for analysis with TJP-AES.

The previous carbonization was used for preparing sea cucumber organisms. First, a 50 mg lyophilized sample was placed into a quartz cup, put into a quartz electric resistance furnace at room temperature, and heated at 400 °C for 40 min. The remainder was weighed, ground in a Plexiglas mortar, and sequentially diluted with a spectroscopic buffer (graphite powder containing 15 wt % NaCl) 10, 100, and 1000 times. A 20 mg diluted sample was introduced into the plasma (3 replicates of each dilution).

Considering the small amount (~3–5 mg) of the protein complex, it was put on 15 mg graphite powder and carbonized at 350 °C for 30 min. Introduction of graphite powder resulted in the reproducibility of 2–5% [11]. The remainder was mixed with graphite powder containing 30 wt % NaCl in a 1:1 ratio and ground in a Plexiglas mortar. The powder obtained was additionally diluted with a spectroscopic buffer 10 times. A 10 mg diluted sample was introduced into plasma (2 and 3 replicates for first and second dilution, respectively). The introduction of diluted organic matrix leads to increasing reproducibility to 3–10% [11]. The results of direct analysis of biological samples by the TJP-AES were confirmed by ICP-OEC [15].

Calibration samples based on graphite powder containing 15 wt % NaCl with an impurity concentration range of 0.01–500 μg/g were used for the construction of calibration curves. These samples were prepared from Russian CRMs of graphite powder with different combinations of impurities (SOG-24, SOG-37, and SOG-21 containing 24, 37, and 21 elements, respectively; Ural Federal University). The calibration samples were stable for at least a year. The final mass percentage of each element was calculated from the difference between experimental and control analyzed powder samples. Control powder consisted of high-purity graphite powder containing 15% NaCl. The powder was used as a blank sample, and low concentrations of Fe and Mg (~0.00002%), which were presented in buffer, were subtracted from the sample concentrations.

The data are presented as micrograms of revealed elements per gram of the sample. The standard deviation of the results did not exceed 5–7%.

The TJP-AES analysis was carried out as in a previous paper [24] using the following run conditions: plasma gas, 4.0 L/min; carrier gas, 0.7 L/min; current strength, 85 A; the angle between jets, 60°; analytical region, 4–5 mm under the jet confluence point. A diffraction spectrograph having a 2400 lines/mm grating covering two spectral ranges (185–350 and 385–470 nm) was used. Spectrum registration was carried out by a photodiode multielement analyzer of emission spectra constructed in Russia by “VMK Optoelektronika”.

### 4.4. Statistical Analysis

The average values of all analyzed samples were estimated using three independent assays for each powder sample analyzed.

## 5. Conclusions

For the first time, we have analyzed and compared the contents of 18 elements in seawater, sea cucumber *E. fraudatrix*, and its very stable protein complex. Only Ca, Mg, B, Sr, Si, and Cr were quantified in seawater in high concentrations, and their content in the whole organisms of cucumbers increased in comparison with seawater (6.9- to 714.5-fold). Compared with seawater, higher concentrations of these elements, except B and Mg, were also obtained in the stable protein complex (9.1- to 120-fold). The contents of other elements (Mn, Cu, Zn, P, As, Ba, Cd, Fe, Mo, Ni, Ag, and Ni), which were not detected in seawater in increased concentrations, were 0.3–1100 µg/g in sea cucumber organisms (except Ni, Ag, and Co) and 0.2–290 µg/g in the protein complex (except Mo). Overall, the data obtained indicate that sea cucumbers strongly accumulate macro- and microelements from seawater.

## Figures and Tables

**Table 1:** Content of various elements in seawater, whole organisms of sea cucumbers *E. fraudatrix,* and its very stable protein complex *.

Element	Content of Elements, μg/g					
	Seawater	Body of the Sea Cucumber	Stable Protein Complex	Ratio of 2 to 1	Ratio of 3 to 1	Ratio of 3 to 2
	1	2	3			
Si	1.5	63.0	180	42.0	120	2.9
Sr	1.7	780	15.0	4.6 × 10^2^	8.8	0.019 (52.0)
Cr	0.13	9.7	4.1	74.6	31.5	0.42 (2.4)
B	5.0	61.0	4.5	13.6	0.9 (1.1)	0.049 (20.3)
Ca	350	2.5 × 10^5^	3200	714.3	9.1	0.0128 (78.1)
Mg	930	6400	44.0	6.9	0.047 (21.1)	0.0068 (145.5)
Mn	n/d (0.005) **	26.0	1.6	>5200	≥320	0.062 (16.3)
Cu	n/d (0.007)	1.4	7.5	≥200	≥1071	5.4
Zn	n/d (0.05)	13.0	51.0	≥260	≥1020	3.9
P	n/d (0.5)	1100	290	≥2200	≥580	0.23 (3.8)
Al	n/d (0.05)	9.3	14.0	≥186	≥280	1.5
Ba	n/d (0.05)	15.0	2.5	≥300	≥50	0.17 (6.0)
Cd	n/d (0.005)	0.3	0.7	≥60	≥140	2.3
Fe	n/d (0.05)	47.0	23.0	≥940	≥460	0.49 (2.0)
Mo	n/d (0.05)	2.8	n/d (2.0)	≥56	-	-
Ni	n/d (0.05)	n/d (1.5)	13.0	-	≥260	-
Co	n/d (0.03)	n/d (1.2)	2.0	-	≥66.6	-
Ag	n/d (0.003)	n/d (0.12)	0.2	-	≥66.6	-

* Mixture of 10 sea cucumbers and stable protein complex obtained from the 10 cucumbers were lyophilized and the content of different elements was determined by two-jet plasma atomic emission spectrometry; the relative standard deviation of the values from three replicates was within 5–7%. ** Not detected, limits of detection are in parentheses.

## Data Availability

All data are given in this article.

## Supplementary Materials

The following supporting information can be downloaded at: https://www.mdpi.com/article/10.3390/molecules27061958/s1.

## Abbreviations

FPLC, fast protein liquid chromatography; TJP-AES, two-jet plasma atomic emission spectrometry; MM, molecular mass.
