# Supplementary material for: Comparison of the Content of Several Elements in Seawater, Sea Cucumber Eupentacta fraudatrix and Its High-Molecular-Mass Multiprotein Complex
Source: Molecules. 2022 Mar 17;27(6):1958. doi: 10.3390/molecules27061958 (PMC8951014; doi:10.3390/molecules27061958)
Supplement: Supplementary file 1 [file molecules-27-01958-s001.zip › molecules-1521201-supplementary.pdf]

molecules

Supplementary File

Comparison of the Content of Several Elements in Seawater, Sea Cucumber *Eupentacta fraudatrix* and Its High-Molecular-Mass Multiprotein Complex

Natalia P. Zaksas [1], Anna M. Timofeeva [2], Pavel S. Dmitrenok [3], Svetlana E. Soboleva [2] and Georgy Nevinsky [2,*]

[1] Institute of Automation and Electrometry, Siberian Division of Russian Academy of Sciences, Pr. Koptyuga 1, Novosibirsk 630090, Russia; natzaksas@gmail.com
[2] Institute of Chemical Biology and Fundamental Medicine, Siberian Division of Russian Academy of Sciences, Pr. Akademika Lavrentieva, 8; Novosibirsk 630090, Russia; bezukaf@mail.ru (A.M.T.); sb543@ngs.ru (S.E.S.)
[3] G. B. Elyakov Pacific Institute of Bioorganic Chemistry; Far Eastern Brunch of the Russian Academy of Sciences, 159 Pr. 100 let Vladivostoku, Vladivostok 690022, Russia; paveldmt@piboc.dvo.ru
* Correspondence: nevinsky@niboch.nsc.ru; Tel.: +7-383-363-51-26; Fax: +7-383-363-51-53

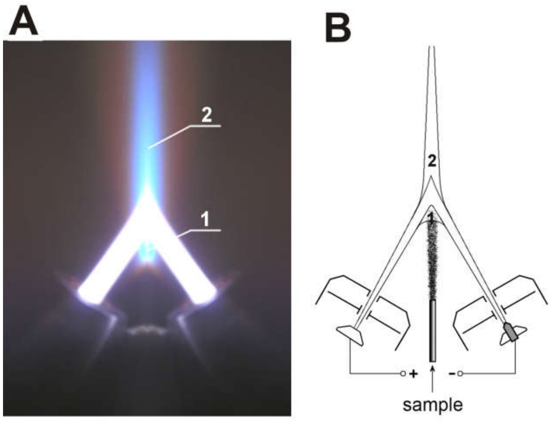

Figure S1. A) Plasma torch; B) electrode unit and analytical regions of the plasma flow: 1, before the jet confluence; 2, after the jet confluence. The figure shows data from the article [30].

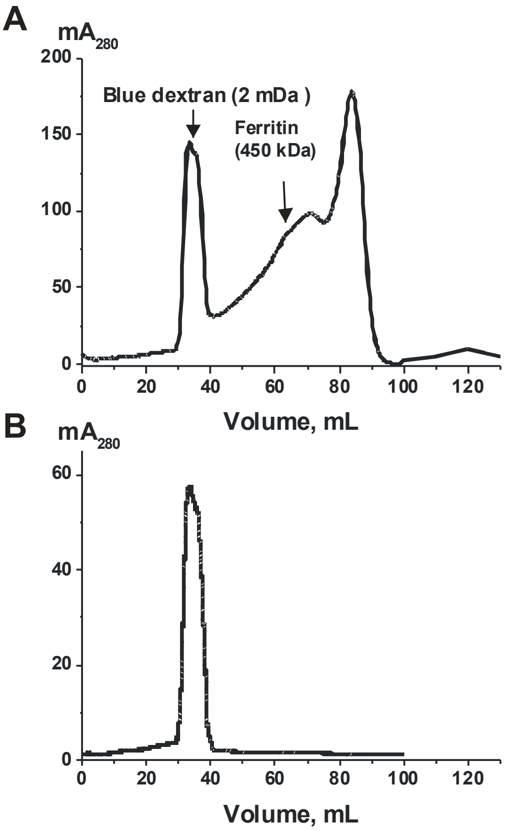

Figure S2. Isolation and analysis of sea cucumber complex. FPLC gel filtration of sea cucumber homogenate proteins on a Sepharose 4B column (**A**). Gel filtration of the purified complex (A) on the Sepharose 4B, ~2000 kDa (**B**). Gel filtration of the complex after its treatment using harsh conditions: 50 mM acidic buffer (pH 2.6), 8.0 M urea, 2 mM DTT, and 1.0 M NaCl (C). In all Panels, (–) - absorbance at 280 nm (A_{280}). The figure shows data from the article [37].

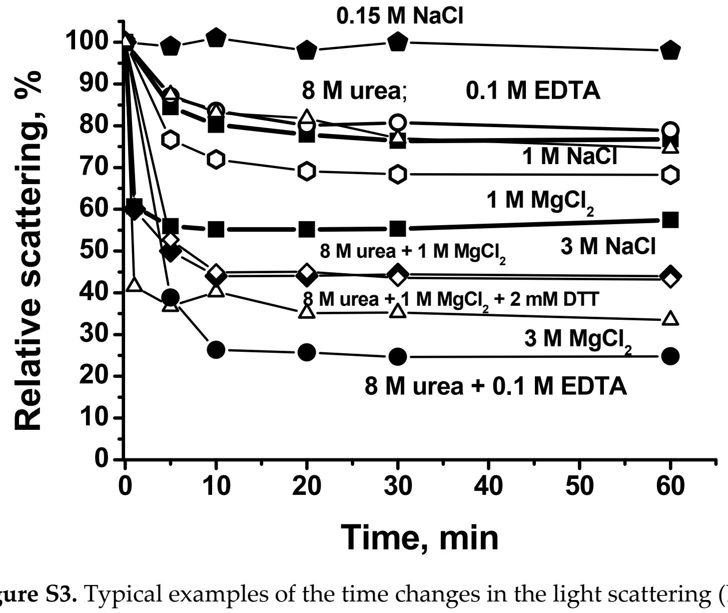

Figure S3. Typical examples of the time changes in the light scattering (LS) of the cucumber complex (0.005 mg/ml) in the presence of 20 mM Tris-HCl buffer (pH 7.5) containing urea, NaCl, $MgCl_2$, DTT, and EDTA in various concentrations and different combinations.
